# Size does matter: 18 amino acids at the N-terminal tip of an amino acid transporter in *Leishmania* determine substrate specificity

**DOI:** 10.1038/srep16289

**Published:** 2015-11-09

**Authors:** Doreen Schlisselberg, Eldar Mazarib, Ehud Inbar, Doris Rentsch, Peter J. Myler, Dan Zilberstein

**Affiliations:** 1 Faculty of Biology, Technion-Israel Institute of Technology, Haifa 32000, Israel; 2 Institute of Plant Sciences, University of Bern, Altenbergrain 21, 3013 Bern, Switzerland; 3 Seattle Biomedical Research Institute, 307 Westlake Ave N, Seattle, WA 98109-5219, USA; 4 Departments of Global Health & Medical Education, University of Washington, Seattle, WA 98195, USA; 5 Departments of Biomedical Informatics & Medical Education, University of Washington, Seattle, WA 98195, USA

## Abstract

Long N-terminal tails of amino acid transporters are known to act as sensors of the internal pool of amino acids and as positive regulators of substrate flux rate. In this study we establish that N-termini of amino acid transporters can also determine substrate specificity. We show that due to alternative *trans* splicing, the human pathogen *Leishmania* naturally expresses two variants of the proline/alanine transporter, one 18 amino acid shorter than the other. We demonstrate that the longer variant (LdAAP24) translocates both proline and alanine, whereas the shorter variant (∆18LdAAP24) translocates just proline. Remarkably, co-expressing the hydrophilic N-terminal peptide of the long variant with ∆18LdAAP24 was found to recover alanine transport. This restoration of alanine transport could be mediated by a truncated N-terminal tail, though truncations exceeding half of the tail length were no longer functional. Taken together, the data indicate that the first 18 amino acids of the negatively charged N-terminal LdAAP24 tail are required for alanine transport and may facilitate the electrostatic interactions of the entire negatively charged N-terminal tail with the positively charged internal loops in the transmembrane domain, as this mechanism has been shown to underlie regulation of substrate flux rate for other transporters.

Parasitic protozoa of the genus *Leishmania* are the causative agents of leishmaniasis, a disease that affects over 12 million people in 88 countries, with ~1.5 million new cases annually. There are three forms of leishmaniasis: cutaneous, mucocutaneous and visceral, the latter, known as kala-azar, is fatal if untreated^1^. *Leishmania* cycle between two major forms in distinct environments: extracellular flagellated promastigotes that proliferate in the alimentary tract of female sand flies; and intracellular spherical, non-flagellated amastigotes that reside in the phagolysosomes of mammalian macrophages^2^. Promastigotes live in a sugar- and amino acid (mostly alanine and proline)-rich, slightly alkaline environment, with a mean temperature of 26 °C. In contrast, amastigotes are exposed to acidic (~pH 5.5), fatty- and amino acid-rich environment at a temperature of 37 °C^3^,^4^. *Leishmania* cells have developed mechanisms of adaptation that favor utilization of amino acids, such as proline and alanine, as alternative carbon sources as well as efficient osmolytes^5^,^6^,^7^,^8^.

Amino acid transporters of *Leishmania* play a critical role in regulating amino acid homeostasis, especially homeostasis of alanine and proline, which are the major constituents of the cellular pool^8^,^9^. We have recently shown that a proline/alanine transporter (LdAAP24) in the plasma membrane of *Leishmania* promastigotes regulates this pool^8^. In the absence of LdAAP24, the cellular pool is empty of proline and the pool of alanine decreases to half that of wild type. Mutants lacking LdAAP24 expression are unable to properly respond to osmotic shock. A similar relationship between amino acid transport and pool regulation has been described previously only for the mammalian neutral amino acid transporter SNAT2. Silencing the expression of SNAT2 in fibroblasts resulted in altered pool content and levels^10^. These two examples raise the possibility that a role for transporters in maintaining intracellular pools of free amino acid is evolutionarily conserved. Presently, the molecular mechanism whereby such transporters regulate the amino acid pools is unclear. We hypothesize that substrate specificity combined with controlled flow rates underlie the mechanism.

A common feature of *Leishmania* amino acid transporters is a long hydrophilic cytoplasmic-facing N-terminus of at least 70 amino acids, most of which belong to the amino acid/auxin permease superfamily^11^,^12^. These long N-termini have strong negative charge, suggesting that they reside at a distance from the negatively charged inner side of the plasma membrane. Further, we consider it likely that such long hydrophilic N-termini interact with the positively charged internal loops of the transporter itself or with neighboring transporters and thereby are involved in regulation of activity. In line with this hypothesis, the *Saccharomyces cerevisiae* general amino acid transporter GAP1, which translocates almost all amino acids, has a very long hydrophilic negatively charged N-terminus of 94 amino acids implicated in self-regulation^13^,^14^,^15^. Specifically, alanine scanning across regions of the intracellular hydrophilic loops and N and C tails has indicated that the last 11 amino acids near the first transmembrane domain are essential for transport activity^16^. However, the role of the other 83 amino acid of GAP1 N-terminal region is not known. The mammalian SNAT2 also comprises a relatively long intracellular N-terminus, but only its short extracellular C-terminus has been shown to have a role in substrate translocation and voltage dependence, without interfering with the transporter’s localization or with binding of substrates to the pocket^17^.

In this work we confirm that in *L. donovani* there are two adjacent genes each encoding for the proline/alanine transporter LdAAP24, but that the two genes are trans-spliced differently. One is trans-spliced upstream to the first AUG of the open reading frame (ORF), giving rise to the full length protein, while the second is trans-spliced 8 bp downstream to the first AUG of the ORF, giving rise to a protein that is shorter by 18 amino acids. We show that the two variants exhibit different substrate specificity: the full length variant translocates both proline and alanine, while the shorter variant transports only proline. In this way, our findings evidence for the first time that the N-terminus indeed plays a role in transport specificity. Furthermore, our data underscore that point mutational analysis alone is likely to be uninformative when investigating self-regulation of transport.

## Results

### The two LdAAP24 isoforms are expressed from two adjacent genes

**Figure 1:**
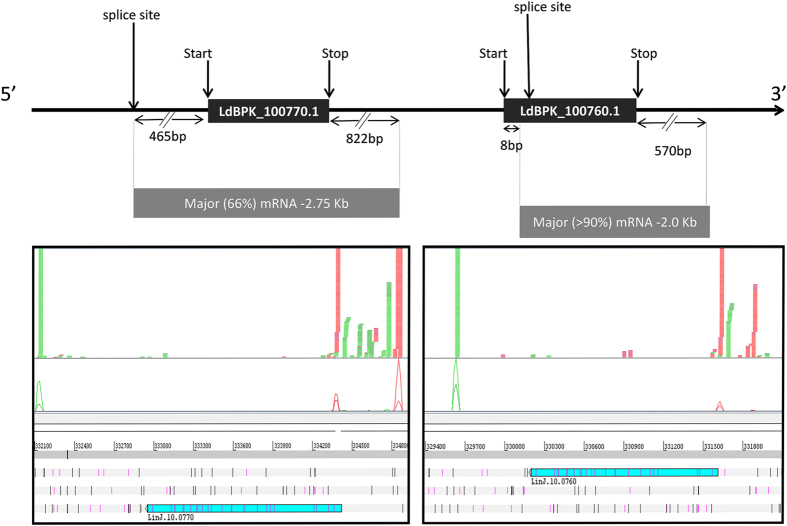
The two LdAAP24 variants are expressed from two adjacent genes. A map describing mRNAs of the two adjacent *LdAAP24* genes. The panels below show reads from SL (red) and polyA (green) RNA-seq libraries mapped to the *L. infantum* genome (see Materials and Methods) and visualized using Artemis^30^. Note that the SL reads mapped to the 2^nd^ (less abundant) site of *LinJ.10.0770* are likely derived from *LinJ.10.0760*, since their sequence is identical at these positions, and Bowtie 2.0 was set to partition non-unique reads randomly between best matches.

In a previous study, we showed that *L. donovani* expresses two variants of the proline/alanine transporter protein, with molecular masses of 42 and 39 kDa^8^. Annotation of the *L. infantum* JPCM5 genome sequence indicates that there are two adjacent gene copies (*LinJ.10.0760* and *LinJ.10.0770*), but both map to a single copy in the current *L. donovani* BPK282A1 genome, suggesting incorrect assembly and/or annotation of this region in the latter. RNA-seq analysis confirmed the presence of both copies in *L. donovani* and revealed that different spliced-leader (SL) and polyadenylation sites were used for the two genes (see Fig. 1). For LdBPK_100770.1, the major SL site was 465 nt upstream of the first AUG, and the major polyadenylation site was 822 nt downstream of the stop codon, resulting in an mRNA of ~2.75-kb. However, for LdBPK_100760.1, the major SL site was located 8 nucleotide (nt) downstream of the first AUG of the annotated CDS, and the polyadenylation site was 570 nts downstream of the stop codon, leading to an ~2.0 kb mRNA. Thus, the two genes generate distinct mRNAs with different 5′ and 3′ UTRs, and the translation initiation site used for LdBPK_100760.1 is likely an AUG 54 nt downstream of that annotated in TriTrypDB. Accordingly, the mRNAs produce two protein isoforms; one (LdBPK_100770.1) from the full-length ORF and the other (LdBPK_100760.1) 18 amino acids shorter.

**Figure 2:**
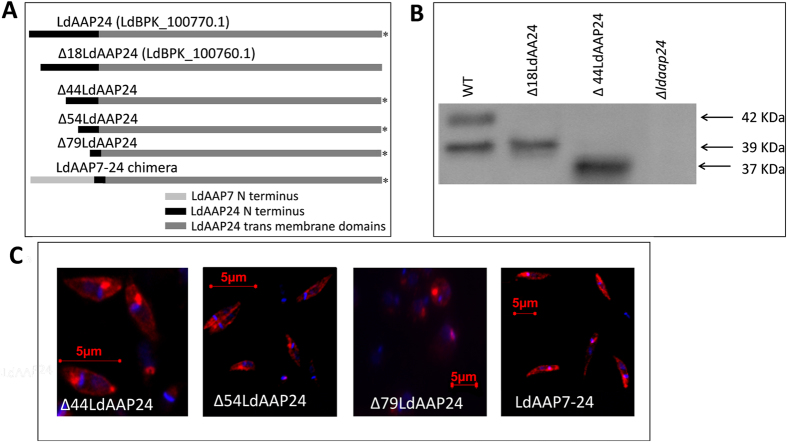
Truncating the hydrophilic N-terminus of LdAAP24 does not affect membrane localization. (**A**) Schematic representation of the different LdAAP24 variants and the length of the N-terminus in each construct. LdAAP24 is the full length variant with an N-terminus of 89 aa; Δ18LdAAP24 has an N-terminus of 71 aa; Δ44LdAAP24 has an N-terminus of 45 aa; Δ54LdAAP24 has an N-terminus of 35 aa; Δ79LdAAP24 has an N-terminus of 10 aa; and LdAAP7-24 has a chimeric N-terminus of 94 aa. Constructs marked with (*) were HA-tagged at the C-terminus. (**B**) Proteins were extracted from the following promastigote strains: WT, LdAAP24-null mutants ectopically expressing Δ18LdAAP24, LdAAP24-null mutants ectopically expressing Δ44LdAAP24, or LdAAP24-null mutant promastigotes (*Δldaap24*). Western blot analysis was performed using anti-LdAAP24 antibody. (**C**) Indirect immunofluorescence of LdAAP24 in LdAAP24-null mutants (*Δldaap24*) ectopically expressing Δ44LdAAP24, Δ54LdAAP24, Δ79LdAAP24 or LdAAP7-24 chimera. Cells were stained with anti-LdAAP24 or with anti-HA antibodies (red) and the DNA stained with DAPI (blue), the latter stains the nucleus and the kinetoplast. The two fluorescent images were merged.

To validate our interpretation, we compared the size of the protein ectopically expressed from an LdAAP24 construct lacking the first 18 amino acids (Δ18LdAAP24) to the wild type LdAAP24 isoform (Figs 2A and S1). The truncated clone was expressed in the *L donovani* proline/alanine null mutant *Δldaap24*. As demonstrated in our earlier study and shown in Fig. 2B, wild type (WT) *L. donovani* promastigotes express two protein variants of LdAAP24, and as predicted, Δ18LdAAP24 aligned with the smaller (39 kDa) variant.

### The two LdAAP24 variants possess distinct transport specificities

**Figure 3:**
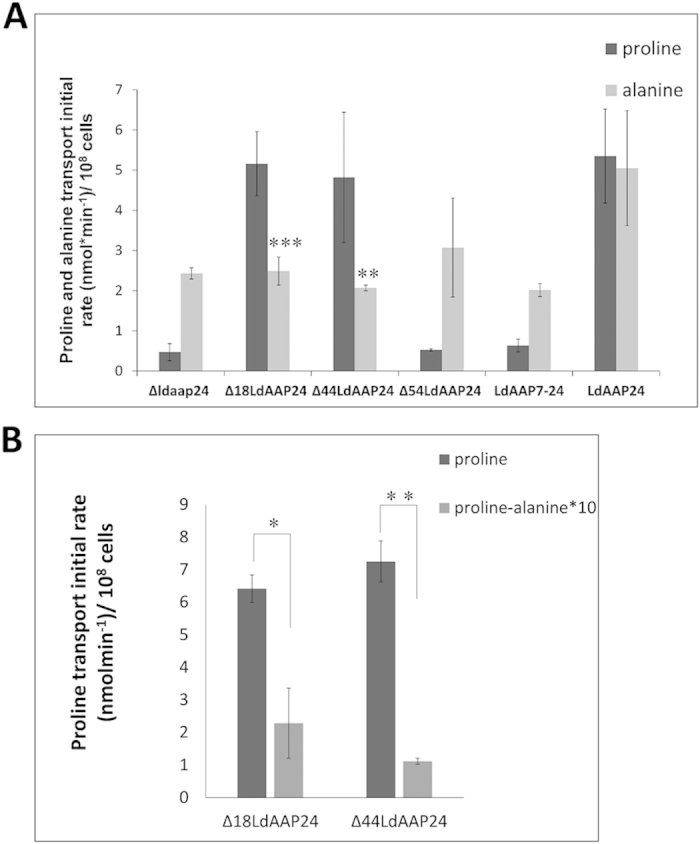
Truncating the hydrophilic N-terminus of LdAAP24 affects substrate translocation activity. (**A**) Initial transport rate (3 minutes) of 1 mM ^3^H L-proline (dark grey) and 1 mM ^3^H L-alanine (light grey) were assayed in the following strains: LdAAP24-null mutants (*Δldaap24*), *Δldaap24* ectopically expressing Δ18LdAAP24, *Δldaap24* ectopically expressing Δ44LdAAP24, *Δldaap24* ectopically expressing Δ54LdAAP24, *Δldaap24* ectopically expressing LdAAP7-24 chimera and *Δldaap24* ectopically expressing full length LdAAP24. Transport was determined at pH 7 and 30 °C. Values represent the mean of at least three independent repeats ± SD. Statistically significant differences (two-tailed T-test) in alanine transport relative to full-length LdAAP24 (p ≤ 0.005) are marked with (**) and (p ≤ 0.001) are marked with (***) (**B**) Initial transport rate (3 minutes) of 1 mM ^3^H L-proline (dark grey) and ^3^H L-proline in the presence of 10-fold concentrations of alanine (light grey) were assayed in *Δldaap24* ectopically expressing Δ18LdAAP24 or Δ44LdAAP24. Transport was determined at pH 7 and 30 °C. Values represent the mean of at least three independent repeats ± SD. Statistically significant differences (p ≤ 0.05) are marked with (*) and (p ≤ 0.005) are marked with (**).

Having established that the two naturally expressed variants of LdAAP24 differ in length, we examined if this truncation impacts translocation specificity. To this end, *Δldaap24* null mutant promastigotes and *Δldaap24* promastigotes expressing full-length LdAAP24 (termed ‘add-back’ strain) or truncated Δ18LdAAP24 were subjected to proline and alanine transport assays. In line with our previous study^8^, null mutants were observed not to take up proline and to transport alanine at half the initial rate of full length add-back (LdAAP24), the latter due to the activity of a second alanine transporter (Fig. 3A). Ectopic expression of full-length LdAAP24 restored both proline and alanine transport (Fig. 3A), although only to the level expected in heterozygous mutants^8^. Notably, promastigotes ectopically expressing Δ18LdAAP24 recovered levels of proline transport similar to the full length add-back, but exhibited no change in alanine transport. The results indicate that the 18 amino acids at the N-terminus are required for alanine transport mediated by LdAAP24.

To further investigate the role of the LdAAP24 N-terminus in transport activity, we constructed several truncations of the 89 hydrophilic amino acids at the N-terminus (Figs 2A,B and S1). Initially, we checked the localization of each truncated transporter. As shown in Fig. 2C, Δ44LdAAP24 and Δ54LdAAP24 proteins localized to the plasma membrane of promastigotes (compare with ectopically expressed full-length LdAAP24 in Fig. 4C in Inbar *et al.*, 2013^8^). In contrast, the transporter with the shortest N-terminus (Δ79LdAAP24) did not reach the plasma membrane; most of it localized near the flagella pocket, likely stuck in the secretory pathway. Next, *Δldaap24* promastigotes ectopically expressing each of these constructs were subjected to proline and alanine transport assays. Δ44LdAAP24 was found to translocate only proline, identical to Δ18LdAAP24 (Fig. 3A). A more extensive truncation of the N-terminus as in Δ54LdAAP24 resulted in ablation of both proline and alanine transport. Of note, even though alanine is not transported by Δ18LdAAP24 and Δ44LdAAP24, it was found still to compete with proline (Fig. 3B), indicating that the N-terminus is necessary for alanine translocation, but not recognition.

To characterize the transport specificity of Δ79LdAAP24, we generated a chimera that should reach the plasma membrane. To this end, we cloned the N-terminus of the lysine transporter, LdAAP7^18^, onto the Δ79LdAAP24 (LdAAP7-24, Fig. 2A). As shown in Fig. 2C, this chimera was indeed observed to locate to the plasma membrane, but nevertheless did not exhibit transport of either proline or alanine (Fig. 3A).

### The N-terminus of the longer LdAAP24 isoform specifically regulates alanine transport

To further analyze the potential role of LdAAP24 N-terminus in alanine and proline transport regulation, the net-charge of the predicted hydrophilic intracellular regions of LdAAP24 was determined (Table 1). As shown, the charge of the N-terminus of LdAAP24 in neutral pH is highly negative (−11.8). Nevertheless, note the positive charge of the last 10 amino acids in the N-terminus adjacent to the first transmembrane domain and the cytosolic loop regions between the transmembrane domains (Δ79LdAAP24, Table 1). Thus, LdAAP24 does still obey the positive-inside rule of trans-membrane proteins^19^,^20^. In light of the highly negative charge of the N-terminus, we propose that a negative electrostatic repulsion occurs towards the inner side of the plasma membrane, resulting in a positive electrostatic interaction between the N-terminus and the internal loops that regulates transport activity. In line with our premise that there is an interaction between the N-terminus and positive intracellular regions of LdAAP24, bioinformatic analysis indicated a disordered structure of the LdAAP24 N-terminus.

**Figure 4:**
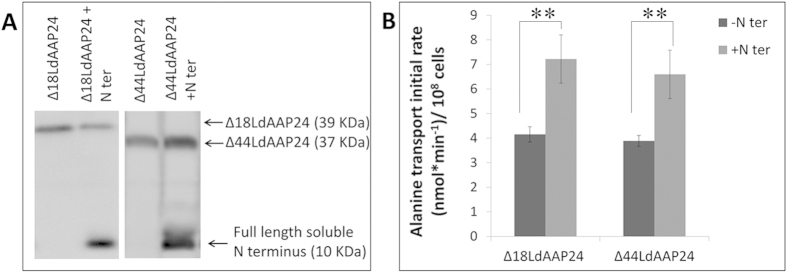
The soluble N-terminus is sufficient to recover alanine transport. (**A**) Western blot analysis of *Δldaap24* ectopically co- expressing Δ18LdAAP24 or Δ44LdAAP24 (39 kDa and 37 kDa, respectively) and the full length N terminus (10 kDa). (**B**) Initial transport rate (3 minutes) of 1 mM ^3^H L-alanine was assayed in LdAAP24-null mutants ectopically expressing Δ18LdAAP24/Δ44LdAAP24 (−N’, dark grey) and LdAAP24-null mutants ectopically co-expressing Δ18LdAAP24/Δ44LdAAP24 and the full length N terminus (+N’, light grey). Transport was determined at pH 7 and 30 °C. Values represent the mean of at least three independent repeats ± SD. Statistically significant differences (p ≤ 0.01) are marked with (**).

To corroborate our hypothesis that the N-terminus serves as a regulatory element, a soluble 89 amino acid N-terminus was ectopically co-expressed with each of the LdAAP24 truncations, and the ability of this soluble N-terminal peptide to impact alanine and proline transport was assessed. Remarkably, co-expression of the soluble N-terminus with Δ18LdAAP24 and Δ44LdAAP24 in promastigotes (Fig. 4A) resulted in full recovery of alanine transport (7 vs. 4 nmol alanine/min/10^8^ cells; Fig. 4B), without any influence on proline transport (Fig. S4).

**Figure 5:**
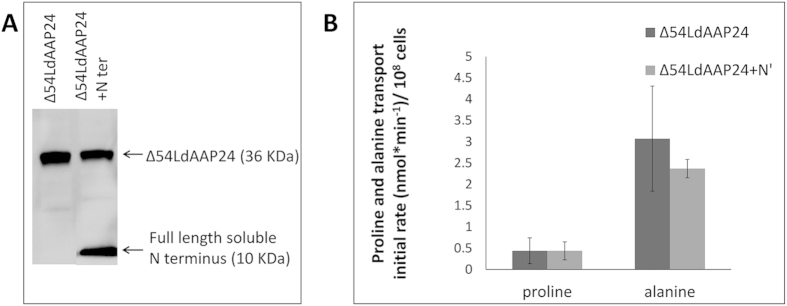
The soluble N-terminus cannot recover proline transport. (**A**) Western blot analysis of *ΔLdAAP24* ectopically co- expressing Δ54LdAAP24 (36 kDa) and the full length N terminus (10 kDa). (**B**) Initial transport rate (3 minutes) of 1 mM ^3^H L-proline or 1 mM ^3^H L-alanine were assayed in LdAAP24-null mutants ectopically expressing Δ54LdAAP24 (−N’, dark grey) and LdAAP24-null mutants ectopically co-expressing Δ54LdAAP24 and the full length N terminus (+N’, light grey). Transport was determined at pH 7 and 30 °C. Values represent the mean of at least three independent repeats ± SD.

These data establish that the N-terminus of LdAAP24 regulates specifically alanine translocation in *Leishmania*. Co-expression of the soluble N-terminus with Δ54LdAAP24 (Fig. 5A) or the LdAAP7-24 chimera (Fig. S2) did not recover proline or alanine transport (Fig. 5B and not shown, respectively). We summarize that truncating more than half of the N-terminus irreversibly impairs the transport activities of LdAAP24.

## Discussion

In this work a new role for the N-terminal of amino acid transporters was established, namely regulation of substrate specificity. To date, two major roles for N-terminal tails have been reported, nutrient sensing and regulation of substrate flux rate^21^,^22^. Here, we show that due to alternative *trans*-splicing, the human pathogen *Leishmania* expresses two variants of the proline/alanine transporter, one variant 18 amino acids shorter than the other. We show that constructs encoding the longer protein, termed LdAAP24, impart proline and alanine transport, whereas constructs encoding the shorter variant, termed ∆18LdAAP24, impart only proline transport, even though ∆18LdAAP24 still recognizes both substrates on the cell surface.

A characteristic feature of LdAAP24, and of most *Leishmania* amino acid transporters, is an 89 amino acid long hydrophilic N-terminus that is negatively charged. The negative charge prompted us to speculate that the N-terminus of LdAAP24 interacts directly or indirectly with the positively charged internal domains of the transporter and thereby, regulates transport activities. Indeed, in the present study we provide support for this hypothesis by demonstrating that co-expression of N-terminally truncated LdAAP24 transporters (Δ18LdAAP24 or with Δ44LdAAP24) with a soluble N-terminus specifically restores alanine transport. Notably, these two truncated transporters although deficient in alanine transport were observed to still recognize alanine, indicating that substrate recognition and transport are distinguishable activities with only transport activity regulated by the18 amino acid N-terminus.

There is a precedent among amino acid transporters for regulatory^21^ interactions between N-termini and internal loops. GAT1, the mammalian GABA transporter has a long hydrophilic N-terminus (52 amino acids long) that positively regulates rate of transport. This regulation is achieved by the negatively charged aspartates within the N-terminus interacting with positively charged arginines within the fourth internal loop of the transmembrane domain (TMD) region. In addition, the N-terminal tail of GAT1 interacts with intracellular proteins such as synaxin1, creating complexes that also regulate the rate of GAT1-mediated GABA transport. As mentioned above, the other previously characterized role for amino acid transporter N-termini is as sensors of internal amino acid pools^22^. A well-studied example is the mammalian neutral amino acid transporter SNAT2 that, in addition to transport, also senses intracellular amino acid availability. Upon amino acid deprivation in L6 myotubes and HeLa cells, SNAT2 protein is stabilized and expression induced via a transcription regulated process. Knockdown assays demonstrate that SNAT2 functions as a mammalian amino acid transceptor that acts in an autoregulatory gene expression pathway. The sensor is localized within the hydrophilic N-terminal region of SNAT2.

It remains unclear how the 18 amino acids at the tip of the LdAAP24 N-terminus regulate substrate specificity. The full-length N-terminus (89 amino acids) is highly negative at physiological pH (−11.8), yet truncating 18 amino acids off its tip only slightly reduces the net charge to −10.9. Further truncation, by half or more, reduces significantly the net charge (up to −5.9) as does replacing the N-terminus entirely with that of another transporter (−4.7) and none of these constructs imparted alanine transport. Indeed, replacing the LdAAP24 N-terminus with the LdAAP7 N-terminus resulted in a construct that no longer imparted transport of proline or alanine. Bioinformatics analysis predicts that both the LdAAP24 and LdAAP7 N-termini are disordered. Taking all our data together, we surmise that the primary structure of the LdAAP24 N-terminus is essential for transport of alanine through the transporter, most likely because it determines the tertiary structure of the transporter and/or interacts with other intracellular regions of the transporter.

Even though physical properties of LdAAP24 N-terminus and cytosolic loops support direct or indirect interaction that regulate transport specificity, we cannot rule out two additional mechanisms; (a) inter-molecular interactions. The soluble N-terminus might interact with other amino acid transporters in *Leishmania*, as suggested by Inbar *et al.*, 2013^8^; or (b) inter-molecular interactions of the soluble N-terminus with cytosolic components, regulating transport through LdAAP24.

Promastigotes reside and proliferate in the mid-gut of sand flies. A common feature of the sand flies’ hemolymph is a high concentration of alanine (around 50 mM), which is much higher than the concentration of proline. In all insects, alanine and proline are the major precursors of metabolic energy, mostly for flight muscles^23^,^24^. Promastigotes are highly adapted to the alanine-rich environment. Promastigotes catabolism resembles that of the insect vector; both alanine and proline are major sources for metabolic energy^6^. However, the metabolic and physiological interplay between the two amino acids, in both vector and parasite, is yet to be discovered. Hence, at this point we speculate that the existence of two natural LdAAP24 variants – one that translocates only proline and the other translocating both proline and alanine - is to balance alanine uptake in the alanine-rich environment of promastigotes.

## Methods

### Materials

^3^H-labelled amino acids were from Amersham. Ampicillin, G418, hygromycin B, phleomycin, M-199 (medium-199) and non-labelled L-amino acids were from Sigma. Fetal bovine serum (FBS) was from Biological Industries. All other reagents were of analytical grade.

### Leishmania cell culture

A clonal line of *L. donovani* 1SR was used in all experiments^25^. To ensure the clonal nature of the cell line, fresh cultures were inoculated using single colonies of promastigotes taken from M-199 agar plates. Promastigotes were grown at 26 °C in M-199 supplemented with 10% FBS.

### Western blot analysis

Western blot analysis was carried out as previously described^9^. Briefly, 10^8^ log phase promastigotes cells were harvested, washed twice in ice-cold phosphate buffered saline (PBS) and re-centrifuged. The resulting pellet was resuspended in Laemmli buffer and then sonicated for 2 s to ensure denaturation. Preparation of salt-extracted membranes was carried out as described in^26^. Total protein was subjected to SDS/PAGE (mini-PROTEAN; Bio-Rad Laboratories) before being transferred to a nitrocellulose membrane. Membranes were blocked with 10% (w/v) non-fat dried skimmed milk powder in PBST (PBS containing 0.1% Tween 20), incubated with anti-LdAAP24 or anti-HA (1:2000 dilution) antibodies for 1 h at room temperature (22 °C), washed and then incubated with secondary goat anti-rabbit HRP (horseradish peroxidase) antibodies (1:10000 dilution).

### Immunofluorescence analyses

For immunofluorescence, mid-log promastigotes were washed twice in PBS and then fixed in 1% formaldehyde/PBS on a slide for 10 min before permeabilization by exposure to 0.2% Triton X-100/PBS for 10 min. Cells were incubated with blocking solution [10% (v/v) non-fat dried skimmed milk powder/PBST] for 30 min at room temperature, incubated with anti-LdAAP24 or anti-HA antibodies (1:200 dilution) for 1 h and then incubated with secondary polyclonal goat anti-rabbit IgG fluorescent antibodies (1:500 dilution; Dy-light 549 Red; Jackson) for 1 h in the dark at room temperature. Finally, cells were washed in PBST and supplemented with 5 μl DAPI (4′,6-diamidino-2-phenylindole; 0.5 μg/ml; Fluka). Fluorescence analyses were carried out using a confocal fluorescent microscope (Axiovert 200M; Zeiss).

### Cloning and expression

PCR-amplified LdAAP24 (open reading frames (ORFs) LinJ10.0760 and LinJ10.0770), as well as truncated versions of the transporter’s ORFs were cloned into *Leishmania* expression vectors pNUS-HnB^27^ between the XhoI and KpnI sites. The soluble N-terminus was cloned into pNUS-HnD^27^ between the XhoI and KpnI sites. All constructs except Δ18LdAAP24 were tagged with an HA-tag at the C-terminus. To make sure that the expected topology of each construct was unaffected, we use TMHMM 2.0 (http://www.cbs.dtu.dk/services/TMHMM/)^28^. Primers for all constructs appear in Table S1. For selection, cells were grown in promastigotes medium containing either phleomycine or blasticidine antibiotics. Successful expression was validated by western blot analysis.

### RNA-sequencing

RNA-seq libraries were prepared using two methods. Splice Leader (SL) RNA-seq libraries, which enrich the 5′ end of mRNAs, were prepared as described elsewhere (http://www.ncbi.nlm.nih.gov/pubmed/?term=23382545). PolyA RNA-seq libraries, which are enriched for the 3′ end of mRNAs, were prepared in a similar fashion, expect that an oligo(dT) primer (TCCGATCTCTTTTTTV) was used for first strand synthesis, a random hexamer primer (TCCGATCTGANNNNNNN) for second strand synthesis and PCR amplification was carried out using seq-primer-CT (AATGATACGGCGACCACCGACACTCTTTCCCTACACGACGCTCTTCCGATCTCT) and R-primer-GA (CAAGCAGAAGACGGCATACGAGCTCTTCCGATCTGA). The libraries were sequenced using the Genome Analyzer IIx (Illumina) at the High Throughput Genomics Unit at the University of Washington to generate 36-nt long single-end reads. Reads were aligned to the *L. donovani* BPK282A1 and *L. infantum* JPCM5 reference genomes in TriTrypDBv5.0 using Bowtie2^29^.

### Transport assays

Transport assays of radiolabeled proline and alanine were carried out as described in^8^. *L. donovani* promastigotes were grown to logarithmic phase, washed twice in ice-cold Earl’s buffer and concentrated to 10^8^ cells/ml. The cell suspension was mixed with reaction mixture (Earl’s buffer, 5 mM glucose, 10 mM Tris and 10 mM succinate) at the relevant pH to a final volume of 600 μl. Cells were then pre-incubated at 30 °C for 10 min. Transport was started by mixing the cell suspension with a specified concentration of non-labelled amino acids and ^3^H L-amino acid substrates in Earl’s buffer at the relevant pH. At 30, 60, 90, 120 and 180 seconds, 100 μl of the mixture was removed and put directly on to 24 mm GF/C glass microfiber filters (Whatman 1822 024). Filters were washed twice in ice cold Earl’s buffer at the relevant pH and soaked in scintillation liquid. Uptake of ^3^H was determined by liquid scintillation counting.

## Additional Information

**How to cite this article**: Schlisselberg, D. *et al.* Size does matter: 18 amino acids at the N-terminal tip of an amino acid transporter in *Leishmania* determine substrate specificity. *Sci. Rep.* **5**, 16289; doi: 10.1038/srep16289 (2015).

## Supplementary Material

Supplementary Information

## Figures and Tables

**Table 1:** Mean charge of constructs’ N-termini.

	Mean charge at pH 7*	pI	Number of AA
LdAAP24 N-terminus	−11.8	4.11	89
Δ18LdAAP24 N-terminus	−10.9	4.02	71
Δ44LdAAP24 N-terminus	−5.9	4.08	45
Δ54LdAAP24 N-terminus	−5.9	4.02	36
Δ79LdAAP24 N-terminus	+1	8.93	10
LdAAP7-24 Chimera N-terminus	−4.7	4.96	95
Loop II	+2	9.7	19
Loop IV	+2	10.1	12
Loop VI	+2	10.1	12
Loop VIII	+4.1	10.1	35

Mean Charge was calculated using Innovagen peptide property calculator.
