# Supplementary material for: Size does matter: 18 amino acids at the N-terminal tip of an amino acid transporter in Leishmania determine substrate specificity
Source: Sci Rep. 2015 Nov 9;5:16289. doi: 10.1038/srep16289 (PMC4637868; doi:10.1038/srep16289)
Supplement: Supplementary Information [file srep16289-s1.pdf]

Size does matter: 18 amino acids at the N-terminal tip of an amino acid transporter in *Leishmania* determine substrate specificity

Doreen Schlisselberg[1], Eldar Mazarib[1], Ehud Inbar[1], Doris Rentsch[2], Peter, J. Myler[3,4,5] and Dan Zilberstein[1*]

Supplementary Information

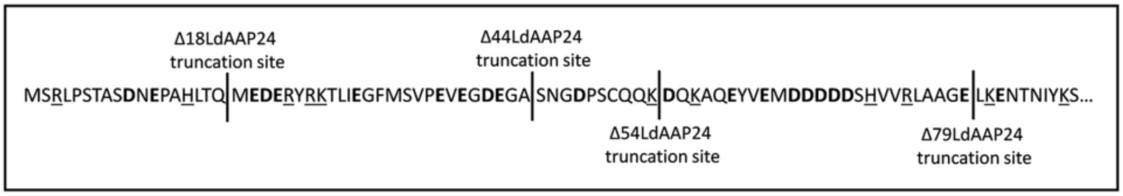

Figure S1. Amino-acid sequence of LdAAP24 N-terminus. Truncation sites for construct preparations are marked.

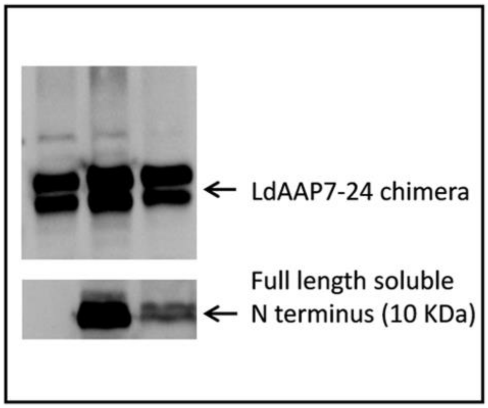

Figure S2. Western blot analysis of *ΔLdAAP24* ectopically co- expressing LdAAP7-24 chimera (43 KDa) and the full length N terminus (10 KDa).

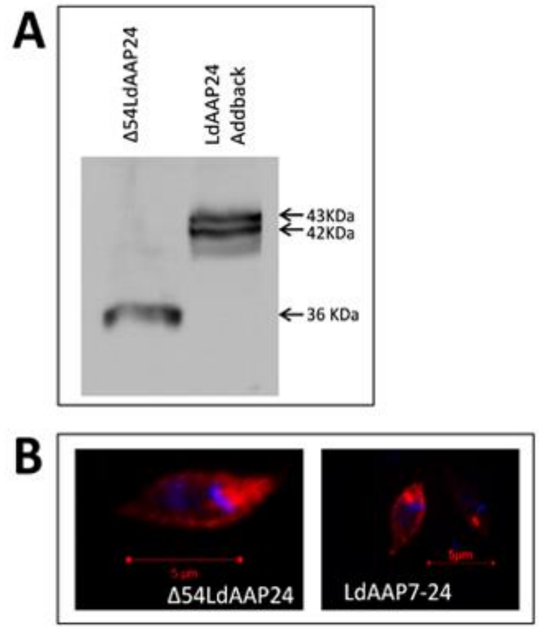

Figure S3. **A.** Western blot analysis on salt-extracted membranes[28] of *ΔLdAAP24* promastigotes ectopically expressing LdAAP24 (42 and 43 KDa) or Δ54LdAAP24 (36 KDa). Polyclonal anti-HA antibodies were used as primary antibodies. **B.** Indirect immunofluorescence of LdAAP24-null mutants (*Δldaap24*) ectopically expressing Δ54LdAAP24 or LdAAP7-24 chimera. Cells were stained with anti-HA antibodies (red) and the DNA stained with DAPI (blue), the latter stains the nucleus and the kinetoplast. The two fluorescent images were merged.

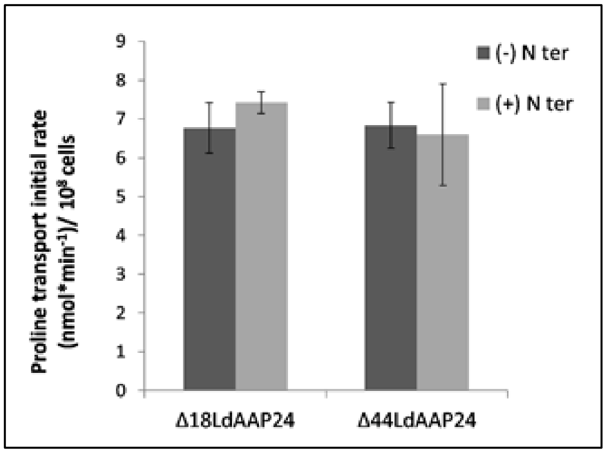

Figure S4. Initial transport rate (3 minutes) of 1 mM ^{3}H L-proline was assayed in LdAAP24-null mutants ectopically expressing Δ18LdAAP24/ Δ44LdAAP24 (-N', dark grey) and LdAAP24-null mutants ectopically co-expressing Δ18LdAAP24/ Δ44LdAAP24 and the full length N terminus (+ N', light grey). Transport was determined at pH 7 and 30 ^{0}C. Values represent the mean of at least three independent repeats ±SD.

Supplementary Table S1. Primer-list.

PCR target	Primer	Restriction enzyme	Plasmid
**LdAAP24 N’**	Fw: AAAGCTCTCGAGATGTCCAGATTGCCAAGCACC<br>Rv: AAAGCTGGTACCCTACTTACCCAGGCGGTTCATT TCGATATCAGTGTAGGACTTGCAGATGTTTGTGTTC	XhoI<br>KpnI	pNUS HnD
**LdAAP24**	Fw: AAAGCTCTCGAGATGTCCAGATTGCCAAGCACC<br>Rv: AAAGCTGGTACCCTATACACTGATATCGAAATG AACCGCCTGGGTAAGGGACTTGCAGATGTTTGTGTTC	XhoI<br>KpnI	pNUS HnB
**Δ18LdAAP24**	Fw: AAAGCT CTCGAGATGGAGGACGAACGTTAC<br>Rv: AAAGCTGGTACCCTATACACTGATATCGAAATG AACCGCCTGGGTAAGGGACTTGCAGATGTTTGTGTTC	XhoI<br>KpnI	pNUS HnB
**Δ44LdAAP24**	Fw: AAAGCTCTCGAGATGAGCAATGGAGACCCCTCC<br>Rv: AAAGCTGGTACCCTATACACTGATATCGAAATG AACCGCCTGGGTAAGGGACTTGCAGATGTTTGTGTTC	XhoI<br>KpnI	pNUS HnB
**Δ54LdAAP24**	Fw: AAAGCTCTCGAGATGGACCAGAAGGCGCAGG<br>Rv: AAAGCTGGTACCCTATACACTGATATCGAAATG AACCGCCTGGGTAAGGGACTTGCAGATGTTTGTGTTC	XhoI<br>KpnI	pNUS HnB
**Δ79LdAAP24**	Fw: ATTATTGGTACCATGACTGTCGTCCGCCT<br>Rv: AAAGCTGGTACCCTATACACTGATATCGAAATG AACCGCCTGGGTAAGGGACTTGCAGATGTTTGTGTTC	XhoI<br>KpnI	pNUS HnB
**LdAAP7-24 chimera**	Fw: ACTTATCTCGAGATGAGCGGCGCTAACCACCAC<br>Split Fw: CATCGAGAGGGTCTACTCCATCATGACTGTCGTCCGCCTCGC<br>Split Rv: GCGAGGCGGACGACAGTCATGATGGAGTAGACCCTCTCGATG<br>Rv: AAAGCTGGTACCCTATACACTGATATCGAAATG AACCGCCTGGGTAAGGGACTTGCAGATGTTTGTGTTC	XhoI<br>KpnI	pNUS HnB
